# LED Lights Influenced Phytochemical Contents and Biological Activities in Kale (*Brassica oleracea* L. var. *acephala*) Microgreens

**DOI:** 10.3390/antiox12091686

**Published:** 2023-08-29

**Authors:** Seom Lee, Chang Ha Park, Jin Kyung Kim, Kyungmin Ahn, Haejin Kwon, Jae Kwang Kim, Sang Un Park, Hyeon Ji Yeo

**Affiliations:** 1 Department of Biological Sciences, Keimyung University, Daegu 42601, Republic of Korea; 2 Department of Microbiology, Keimyung University School of Medicine, Daegu 42601, Republic of Korea; 3 Department of Statistics, Keimyung University, Daegu 42601, Republic of Korea; 4 Department of Crop Science, Chungnam National University, Daejeon 34134, Republic of Korea; 5 Division of Life Sciences, College of Life Sciences and Bioengineering, Incheon National University, Yeonsu-gu, Incheon 22012, Republic of Korea; 6 Department of Smart Agriculture Systems, Chungnam National University, Daejeon 34134, Republic of Korea; 7 Biological Resource Center, Korea Research Institute of Bioscience and Biotechnology (KRIBB), Jeongeup 56212, Republic of Korea

**Keywords:** kale microgreens, LED lights, glucosinolate, carotenoid, phenolics, antioxidant effect, antibacterial effect

## Abstract

Light-emitting diodes (LEDs) are regarded as an effective artificial light source for producing sprouts, microgreens, and baby leaves. Thus, this study aimed to investigate the influence of different LED lights (white, red, and blue) on the biosynthesis of secondary metabolites (glucosinolates, carotenoids, and phenolics) and the biological effects on kale microgreens. Microgreens irradiated with white LEDs showed higher levels of carotenoids, including lutein, 13-*cis*-β-carotene, α-carotene, β-carotene, and 9-*cis*-β-carotene, than those irradiated with red or blue LEDs. These findings were consistent with higher expression levels of carotenoid biosynthetic genes (*BoPDS* and *BoZDS*) in white-irradiated kale microgreens. Similarly, microgreens irradiated with white and blue LEDs showed slightly higher levels of glucosinolates, including glucoiberin, progoitrin, sinigrin, and glucobrassicanapin, than those irradiated with red LEDs. These results agree with the high expression levels of *BoMYB28-2*, *BoMYB28-3*, and *BoMYB29* in white- and blue-irradiated kale microgreens. In contrast, kale microgreens irradiated with blue LEDs contained higher levels of phenolic compounds (gallic acid, catechin, ferulic acid, sinapic acid, and quercetin). According to the total phenolic content (TPC) and 2,2-diphenyl-1-picrylhydrazyl (DPPH) inhibition assays, the extracts of kale microgreens irradiated with blue LEDs had slightly higher antioxidant activities, and the DPPH inhibition percentage had a positive correlation with TPC in the microgreens. Furthermore, the extracts of kale microgreens irradiated with blue LEDs exhibited stronger antibacterial properties against normal pathogens and multidrug-resistant pathogens than those irradiated with white and red LEDs. These results indicate that white-LED lights are suitable for carotenoid production, whereas blue-LED lights are efficient in increasing the accumulation of phenolics and their biological activities in kale microgreens.

## 1. Introduction

Kale (*Brassica oleracea* var. *acephala*) is regarded as one of the most important Brassica crops worldwide because of its use in salads, beverages, and cuisine [1]. It has been shown to possess strong biological activities, such as antioxidant [2], antibacterial [3], and antiproliferative effects [4]. These activities may be derived from health-beneficial functional compounds belonging to the classes of glucosinolates, phenylpropanoids, flavonoids, phenolic acids, and carotenoids [5]. Microgreens are plant seedlings that are older than sprouts but younger than baby leaves and are characterized by high concentrations of bioactive components [6]. Brassica microgreens usually contain a higher nutrient and phytochemical content than grown plants [7]. Therefore, the consumption of kale microgreens can improve human health.

Glucosinolates are secondary metabolites that are specifically distributed in Brassica plants and can be subdivided into three groups (aromatic, aliphatic, and indolic) based on three different initial precursors (tryptophan, methionine, and phenylalanine) [8]. The MYB28, MYB29, and MYB76 transcription factors are reported to regulate aliphatic glucosinolate biosynthesis [9], as are the other MYB transcription factors (MYB34, MYB51, and MYB122), which are known to be involved in indolic glucosinolate biosynthesis [10]. Furthermore, these biosynthesized glucosinolates can be hydrolyzed into epithionitriles, nitriles, oxazolidinethiones, substituted isothiocyanates, and thiocyanates [11], and the resulting hydrolysis products exert antimicrobial effects against pathogens [12].

Carotenoids are pigments that result in yellow, orange, and red colors and can be subdivided into two groups (oxygenated and hydrocarbon groups) [5]. Approximately 50 carotenes, including α-carotene, β-carotene, and lycopene, are hydrocarbons, and approximately 800 xanthophylls, including lutein, β-cryptoxanthin, astaxanthin, and zeaxanthin, are carotenoids with oxygen atoms [13]. Carotenoid biosynthesis begins with the condensation of two molecules of geranylgeranyl diphosphate through the catalysis of phytoene synthase (PSY). Phytoene can then be converted to lycopene by phytoene desaturase (PDS) and ζ-carotene desaturase (ZDS). From lycopene, the carotenoid biosynthesis pathway can split into the α-branch and the β-branch. Then, α-carotene and lutein can be biosynthesized from α-branch, as well as β-carotene, β-cryptoxanthin, and zeaxanthin, which can be generated from β-branch [14]. Carotenoids are not biosynthesized in animals; thus, they only gain these phytochemicals from dietary sources [5]. These metabolites are beneficial for enhancing human health (e.g., eyes) and preventing human diseases (e.g., cancers) [5]. Kale microgreens may be considered a good source of dietary carotenoids because carotenoids, which are distributed in mature kale, are also found in high amounts in microgreens.

Phenolic compound groups are metabolites derived from phenylalanine and are widely distributed in higher plants. These molecules possess biological activities (anticancer, antimicrobial, antiestrogenic, and antioxidant properties) [5]. Therefore, consumption of edible plant products containing high amounts of phenolic compounds is recommended for health promotion.

Light-emitting diodes (LEDs) have been used in plant factories and indoor gardening systems because they are effective sources for enhancing microgreen production and phytochemical accumulation owing to their longevity, durability, wavelength diversity, and size [15]. Previous studies have reported that LED light positively affects the production of many functional compounds, including glucosinolates, phenolics, and carotenoids, in the sprouts or microgreens of *Brassica* plants. Therefore, the aim of this study was to optimize different LED light wavelengths (blue (470 nm), red (660 nm), or white (380 nm)) for the production of microgreens and the accumulation of carotenoids, phenolics, and glucosinolates in kale. In addition, this study provides information on the synergistic antioxidant and antibacterial activities of secondary metabolites from kale microgreen. In particular, this is a novel study proving that the extracts of kale microgreens possess an antibacterial effect against multidrug-resistant *Pseudomonas aeruginosa*.

## 2. Materials and Methods

### 2.1. Plant Materials and Growth Conditions

Kale seeds were obtained from Asia Seed Co. Ltd. (Seoul, Republic of Korea). For seed germination, 100 seeds were immersed in autoclaved distilled water for 24 h and placed in a plastic pot containing vermiculite. Kale microgreens were grown in a growth chamber at 25 °C and irradiated with LED lights (white (450–660 nm), blue (450 nm), or red (660 nm)) with a flux rate of 90 μmol·m^−2^·s^−1^ and a long-day photoperiod (16 h light/8 h dark cycle). The PARUS LED light (PARUS LED Co., Cheoan, Republic of Korea) comprised white-, red-, and blue-light components. One pot including 100 seeds represented one biological replicate, and three biological replicates were utilized in the present study. Microgreens were harvested after 10 days of LED lighting treatment. These samples of kale microgreen were then frozen in liquid nitrogen directly after harvesting and freeze-dried at −80 °C. The samples were then ground into fine powders for further HPLC analysis of glucosinolates and phenolic compounds.

### 2.2. Quantitative Real-Time Polymerase Chain Reaction

Total RNA was isolated from kale microgreens irradiated with white-, blue-, and red-LED light using the TRIzol method and an RNA extraction kit (Geneaid, Sijhih, Taiwan). Next, cDNA was synthesized from 1 μg of the extracted RNA using the PrimeScript 1st strand cDNA Synthesis Kit (Dakara, Seoul, Republic of Korea). The resulting cDNA was diluted 20-fold, followed by PCR amplification using a CFX96 real-time system with a C1000 thermal cycler (Bio-Rad, Hercules, CA, USA). Each assay was performed with 20 μL of reaction mix consisting of 5 μL of cDNA, 3 μL of nuclease-free water, 1 μL of each specific primer (0.5 μM), and 10 μL of 2× Real-Time PCR Master Mix kit with SFCgreen I (BioFACT, Daejeon, Republic of Korea) (Tables S1 and S2). The expression levels of the carotenoid and glucosinolate biosynthesis genes (Table S3) were calculated using the relative quantification method [16].

### 2.3. Extraction of Carotenoid

Carotenoid analysis of kale microgreens grown under LED irradiation was performed according to a previously reported method [5]. Briefly, 100 mg of freeze-dried kale microgreens radiated with red-, blue-, and white-LED light were mixed with 0.1% ascorbic acid/ethanol (3 mL, *w*/*v*), followed by vortexing for 30 s and incubation at 85 °C for 5 min. Potassium hydroxide (120 µL, 80% *w*/*v*) was added to remove any interfering oils and then immediately incubated on ice for 10 min. β-apo-8′-carotenal (100 μL, 25 ppm) was added as an internal standard, and HPLC-grade water (1.5 mL) was added. Afterward, hexane (1.5 mL) was added, and the sample was centrifuged at 1200× *g* and 85 °C. The upper layer was transferred to a new tube. The residual lower layer was extracted twice with hexane (1.5 mL). The collected layers were dried using nitrogen gas. Dichloromethane/methanol (50/50 *v*/*v*) was used for resolution, and the resulting extract was transferred to a vial.

### 2.4. Analysis of Carotenoid

A total of 10 μL of the syringe-filtered extracts were injected on an Agilent Technologies 1100 HPLC series (Palo Alto, CA, USA) equipped with a PDA detector. YMC column (250 × 4.6 mm, 3 μm; Waters Corporation, Milford, MA, USA) controlled at 450 nm and 40 °C was used to isolate individual carotenoids. The gradient program was employed as previously described [5]: eluent A, methyl tert-butylether; eluent B, 92% methanol with 10 mM ammonium acetate; 0 min, 90% B; 20 min, 83% B; 29 min, 75% B; 35 min, 30% B; 40 min, 30% B; 42 min, 25% B; 45 min, 90% B; and 55 min, 90% B with a flow rate of 1.0 mL/min. Identification of five carotenoids was carried out and compared with retention time and co-elution of *β-apo-8′-carotenal* (Figure S1), as well as quantification of lutein (≥95%), 13-*cis*-β-carotene (≥95%), α-carotene (≥95%), β-carotene (≥95%), and 9-*cis*-β-carotene (≥95%) according to each calibration curve. The linear equations were y = 0.1847x + 0.1214 for lutein, y = 0.2154x + 0.1814 for β-carotene, y = 0.4284x + 0.0339 for 9-*cis*-β-carotene, y = 0.3361x − 0.0220 for 13-*cis*-β-carotene, and y = 0.5479x − 0.0805 for α-carotene. The chemical standards were purchased from CaroteNature (Lupsingen, Switzerland).

### 2.5. Extraction of Desulfo-Glucosinolates

Desulfo-glucosinolates from kale microgreens grown under irradiation with LED light were extracted according to a previously reported method [5]. Briefly, 100 mg of freeze-dried kale microgreens radiated with red-, blue-, and white-LED light were mixed with 0.5 mL of 70% methanol (*v*/*v*). The extract was ultrasonicated at 70 °C for 5 min to inactivate the endo-myrosinases and then centrifuged at 4 °C and 11,000× *g* for 20 min. This procedure was repeated twice to obtain the crude extract. Subsequently, a mini-column was made of DEAE-Sephadex A-25 (H + form by 0.5 M sodium acetate). The collected extracts were loaded onto a mini-column and washed with HPLC-grade water, followed by the addition of an arylsulfatase solution (75 µL). After 16 h of incubation at 25 °C, desulfo-glucosinolates were eluted with 0.5 mL of HPLC grade water three times and then filtered via a 0.45 µm hydrophilic PTFE syringe filter (Futecs Co., Ltd., Daejeon, Republic of Korea). DEAE-Sephadex A-25 and arylsulfatase were purchased from Sigma-Aldrich, St. Louis, MO, USA.

### 2.6. Analysis of Desulfo-Glucosinolates

A total of 10 μL of the syringe-filtered extracts were injected on an Agilent Technologies 1200 HPLC series (Palo Alto, CA, USA) equipped with a PDA detector. Inertsil ODS-3 columns (150 × 3.0 mm, 3 μm; GL Sciences, Tokyo, Japan) and an E-type cartridge guard column (10 × 2.0 mm i.d., 5 µm) controlled at 227 nm and 40 °C were used to isolate desulfo-glucosinolates. The gradient program was employed as previously described [5,17]: eluent A, water; eluent B, acetonitrile; 0–18 min, 7–24% B; 18–32 min, 24% B; 32.01 min, 7% B; and 32.01–40 min, 7% B with a flow rate of 0.2 mL/min. The quantification of glucoiberin, progoitrin, glucoraphanin, sinigrin, glucobrassicanapin, and glucoerucin was carried out as previously described [5,17].

### 2.7. Extraction of Phenolics

Phenolic compound analysis of kale microgreens grown under LED irradiation was performed according to a previously reported method [5]. Briefly, 100 mg of freeze-dried kale microgreens radiated with red-, blue-, and white-LED light were mixed with 1 mL of 70% methanol (*v*/*v*). The extract was ultrasonicated at 35 °C for 60 min and then centrifuged at 4 °C and 11,000× *g* for 20 min. The crude extract was loaded onto a 0.45 µm hydrophilic PTFE syringe filter.

### 2.8. Analysis of Phenolics

A total of 50 μL of the syringe-filtered extracts were injected on a Futecs NS-4000 system (Daejeon, Republic of Korea) equipped with a UV detector. OptimaPak column (250 × 4.6 mm, 5 µm; RStech Co., Daejeon, Republic of Korea) controlled at 280 nm and 30 °C was used to isolate individual phenolic compounds. The gradient program was employed as previously described [5]: eluent A, methanol; eluent B, acetic acid–water (0.2% *v*/*v*); 0 min, 95% B; 4 min, 95–85% B; 9 min, 85% B; 14 min, 85–80% B; 24 min, 80% B; 54 min, 80–70% B; 55 min, 70–55% B; 65 min, 55% B; 75 min, 55–44% B; 77 min, 44–40% B; 79 min, 40% B; 80 min, 40–20% B; 90 min, 20% B; 91 min, 20–95% B; and 98 min, 95% B with a flow rate of 1.0 mL/min. Identification of six phenolics was carried out and compared with retention time and spike test (Figure S1), as well as quantification of gallic acid (≥99%), catechin (≥98%), ferulic acid (≥99%), sinapic acid (≥98%), rutin (≥99%), and quercetin (≥95%) according to each calibration curve. The linear equations were y = 32.89591693x − 26.17370908 for gallic acid, y = 7.889742787x − 40.24235366 for catechin, y = 38.06733632x + 115.7556042 for ferulic acid, y = 16.97022716x + 7.311514962 for sinapic acid, y = 8.09714215x − 105.546569 for rutin, and y = 14.00604622x − 148.3452191 for quercetin. The chemical standards were purchased from Sigma-Aldrich (Seoul, Republic of Korea).

### 2.9. Total Phenolic Content

Using the syringe-filtered extracts, the total phenolic contents were assessed as previously described [18]. A total of 3.4 mL of HPLC-grade water, 0.5 mL of Folin and Ciocalteu’s phenol reagent (2N), and 0.1 mL of each extract from kale microgreens grown under LED irradiation were mixed and allowed to stand for 5 min. After the addition of sodium carbonate (20% *w*/*v*), the mixture was incubated in the dark for 1 h, and then absorbance was measured at 760 nm. The total phenolic content of kale microgreens grown under LED irradiation was expressed as gallic acid equivalents. The linear equation was y = 0.000076 x − 0.077087 (R^2^ = 0.997).

### 2.10. Measurement of Antioxidant Activity

The free radical 2,2-diphenyl-1-picrylhydrazyl (DPPH) is generally used to evaluate the in vitro antioxidant activity of components from kale microgreens grown under irradiation with LED light for 10 days using a previously reported method [19]. Briefly, 1.9 mL of a 0.3 mM DPPH ethanol solution and 0.1 mL of extracts were added to 2 mL tubes and incubated for 15 min in the dark. The absorbance of DPPH was measured at 524 nm. The following equation was used: DPPH scavenging activity (%) = [(1 − A1)/A0] × 100, where A0 is the absorbance of the control and A1 is the absorbance of the kale microgreen sample.

### 2.11. Antibacterial Screening of Kale Microgreens

Antibacterial screening of kale microgreens cultivated under different LED lights for 10 days was performed using disc diffusion [19]. Kale microgreen powder (150 mg) was added to 30 mL of methanol and vortexed for 10 s, followed by shaking at 120 rpm for 24 h. After filtering the extract through filter paper, evaporation was performed in a rotary vacuum evaporator. The evaporated extract was dissolved in dimethylsulfoxide (DMSO) at a final concentration of 100 mg/mL. The human pathogens *Bacillus cereus* (KCTC 3624), *Escherichia coli* (KCTC 1682), *E*. *Coli* (PVC19), *Pseudomonas aeruginosa* (KCCM 11803), *Staphylococcus aureus* (KCTC 3881), *Micrococcus luteus* (KCTC 3063), *Staphylococcus epidermidis,* and *P*. *aeruginosa* (1113), *P*. *aeruginosa* (1828), *P*. *aeruginosa* (1731), *P*. *aeruginosa* (0225), *P*. *aeruginosa* (0826), *P*. *aeruginosa* (1378), *P*. *aeruginosa* (p01827) were pre-cultured in nutrient broth overnight in a shaker at 37 °C to OD600 = 0.5 in each medium. Before pouring the warm medium into the Petri dishes, an aliquot (100 µL) of each bacterial culture solution was added, and the medium was solidified. Thereafter, 60 µL of each extract was applied to a sterile paper disk, and three sterile paper disks were placed on agar plates. Each disk contained 6 mg of extract derived from kale microgreens grown under different LED lights for 10 days. Each disk was incubated at 37 °C for 24 h, and the inhibition zones were measured.

### 2.12. Statistical Analysis

Duncan’s multiple range test for data from glucosinolate, carotenoid, and phenolic compound HPLC analyses, total phenolic content, and DPPH assays was performed using SAS software version 9.2 (SAS Institute Inc., Cary, NC, USA).

### 2.13. Chemicals

Acetonitrile, methanol, ethanol, acetic acid, hexane, methyl tert-butylether, dichloromethane, water, potassium hydroxide were purchased from Daejung, Siheung, Korea. Folin and Ciocalteu’s phenol reagent, β-apo-8′-carotenal, 2,2-diphenyl-1-picrylhydrazyl, and dimethylsulfoxide were purchased from Sigma-Aldrich, St. Louis, MO, USA.

## 3. Results

### 3.1. Carotenoid Biosynthesis Gene Expression Analysis in Kale Microgreens Irradiated with White-, Blue-, and Red-LED Lights

**Figure 1:**
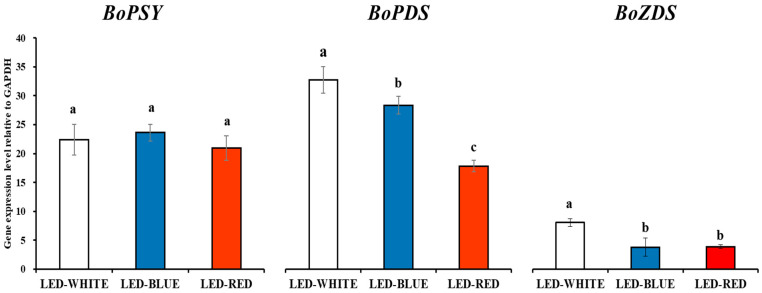
Expression of carotenoid biosynthesis genes in kale microgreens irradiated with white-, blue-, and red-LED lights. Means with the different letters (a–c) are significantly different at *p* < 0.05 using DMRT.

qRT-PCR was carried out using kale microgreens irradiated with white-, blue-, and red-LED lights, using *BoGAPDH* as a reference gene for normalization. The expression levels of three carotenoid biosynthesis genes, *BoPSY*, *BoPDS*, and *BoZDS*, were evaluated using qRT-PCR. The expression levels of *BoPSY* did not differ significantly. However, the expression levels of *BoPDS* and *BoZDS* were statistically higher in white-irradiated kale microgreens, followed by blue- and red-irradiated kale microgreens (Figure 1).

### 3.2. Carotenoid Contents in Kale Microgreens Irradiated with White-, Blue-, and Red-LED Lights

Five carotenoids (lutein, 13-*cis*-β-carotene, α-carotene, β-carotene, and 9-*cis*-β-carotene) were detected in the microgreens of kale and analyzed using HPLC. β-carotene showed the greatest abundance among the five carotenoids in kale microgreens. White-LED-radiated microgreens contained significantly higher levels of 13-*cis*-β-carotene, α-carotene, β-carotene, and 9-*cis*-β-carotene than microgreens under red- or blue-LED lights. However, white- and red-LED treatments resulted in higher levels of lutein than microgreen plants under blue-LED light (Table 1). These findings were consistent with the expression levels of *BoPSY*, *BoPDS*, and *BoZDS* (Figure 1).

### 3.3. Alipathic Glucosinolate Biosynthesis Involved Tion Factor Expression Analysis in Kale Microgreens Irradiated with White-, Blue-, and Red-LED Lights

**Figure 2:**
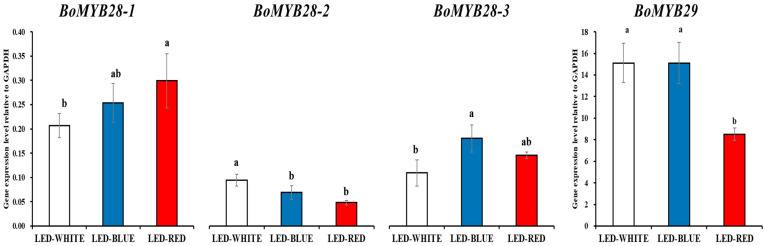
Expression of alipathic glucosinolate biosynthesis involves transcription factors in kale microgreens irradiated with white-, blue-, and red-LED lights. Means with the different letters (a or b) are significantly different at *p* < 0.05 using DMRT.

The expression levels of the four biosynthesis genes, *BoMYB28-1*, *BoMYB28-2*, *BoMYB28-3*, and *BoMYB29*, were evaluated using qRT-PCR. The expression levels of *BoMYB28-2* were significantly higher in white-irradiated kale microgreens, whereas the expression levels of *BoMYB29* were significantly higher in white- and blue-irradiated kale microgreens. The expression levels of *BoMYB28-1* and *BoMYB28-3* were the highest in the red- and blue-irradiated kale microgreens, respectively (Figure 2).

### 3.4. Alipathic Glucosinolate Contents in Kale Microgreens Irradiated with White-, Blue-, and Red-LED Lights

We identified that kale microgreens contained six glucosinolates (including glucoiberin, progoitrin, glucoraphanin, sinigrin, glucobrassicanapin, and glucoerucin). LED lighting treatments significantly improved glucosinolate production. Among the 11 glucosinolates detected, progoitrin was present in the highest concentration in kale microgreens. The highest levels of glucoraphanin were observed in blue- and red-LED-irradiated microgreens. White- and red-LED treatments resulted in higher levels of glucoiberin, whereas white and blue-LED treatments resulted in higher levels of progoitrin, glucobrassicanapin, and sinigrin (Table 2). These findings agree with the expression levels of *BoMYB28-1*, *BoMYB28-2*, *BoMYB28-3*, and *BoMYB29* (Figure 2).

### 3.5. Phenolic Contents in Kale Microgreens Irradiated with White-, Blue-, and Red-LED Lights

Six phenolic compounds (gallic acid, catechin, ferulic acid, sinapic acid, rutin, and quercetin) were detected and quantified using the spike test, retention time, and external standard calibration curves for kale microgreens. The highest levels of gallic acid, catechin, ferulic acid, sinapic acid, and quercetin were observed in blue-LED-irradiated microgreens grown for 10 days, followed by microgreens grown under white-LED lights and microgreens grown under red-LED lights (Table 3).

### 3.6. Total Phenolic Content and DPPH Assay

The total phenolic content was measured in kale microgreens grown under different LED lights. These results show that kale microgreens exposed to blue-LED radiation had the highest phenolic content. The DPPH radical scavenging activity of kale microgreens was evaluated using extracts of kale microgreens grown under different LED lights. All three kale microgreen treatments exhibited powerful antioxidant effects. Among them, blue-LED-irradiated kale had the best antioxidant ability. DPPH radical inhibition was positively correlated with the phenolic components capable of antioxidant activity (Table 4).

### 3.7. The Antimicrobial Effect of Kale Microgreens Irradiated with White-, Blue-, and Red-LED Lights

**Figure 3:**
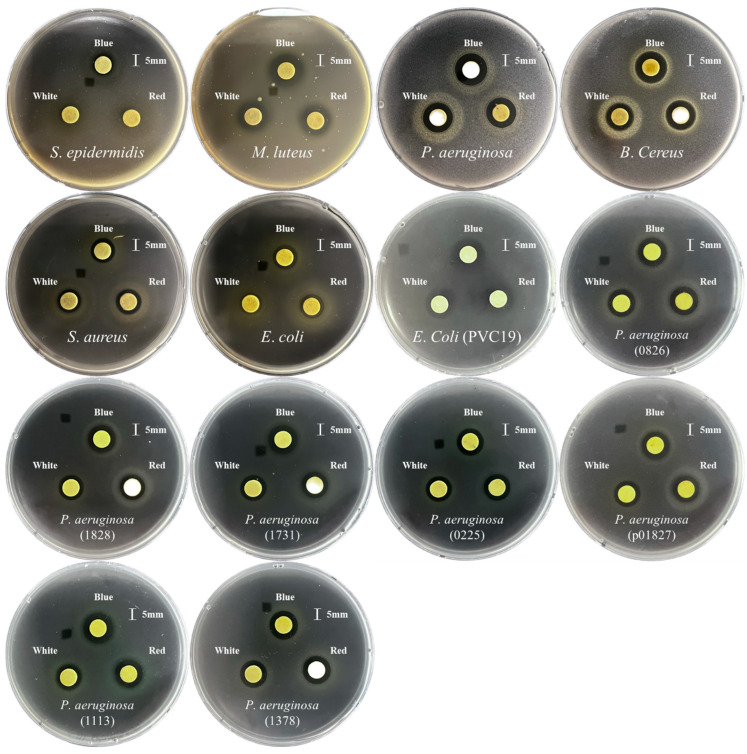
Representative images showing antibacterial activities of methanol extracts of kale microgreens grown under different LED light illuminations (left, extracts of kale microgreens grown under white-LED light; middle, extracts of kale microgreens grown under blue-LED lights; right, extracts of kale microgreens grown under red-LED lights).

The antimicrobial effects of kale microgreens grown under different LED lights were tested against a wide variety of microorganisms, including Gram-negative, Gram-positive, and multidrug-resistant pathogens (Figure 3 and Table S4). The antimicrobial effects of extracts of blue-LED-irradiated kale microgreens were most powerful against pathogens, which may be due to the higher amount of total phenolic components. The growth rates of *B*. *cereus* (KCTC 3624), *E. coli* (KCTC 1682), *E*. *Coli* (PVC19), *P. aeruginosa* (KCCM 11803), *S. aureus* (KCTC 3881), *M. luteus* (KCTC 3063), *S*. *epidermidis,* and *P*. *aeruginosa* (1113), *P*. *aeruginosa* (1828), *P*. *aeruginosa* (1731), *P*. *aeruginosa* (0225), *P*. *aeruginosa* (0826), *P*. *aeruginosa* (1378), and *P*. *aeruginosa* (p01827) were effectively inhibited, particularly by extracts of blue-LED-radiated kale microgreens.

## 4. Discussion

*Brassica* vegetables are good sources of glucosinolates, phenolics, and carotenoids that are beneficial for human health. In this study, five carotenoids, five phenolics, and ten glucosinolates were detected in kale microgreens grown under different LED lights. These results are consistent with those of previous studies that analyzed glucosinolates, phenolics, and carotenoids. According to previous studies, kale sprouts contain glucoiberin, progoitrin, glucoraphanin, sinigrin, and glucoerucin [20]. In addition, kale microgreens contain glucoiberin, sinigrin, and glucoibervirin [21]. Waterland et al. (2019) reported a high abundance of lutein, β-carotene, neoxanthin, violaxanthin, antheraxanthin, α-carotene, and zeaxanthin in kale microgreens. In particular, the total carotenoid levels in microgreen 1 (kale with two fully expanded cotyledons), microgreen 2 (kale with two fully expanded true leaves), baby leaf 1 (kale with four fully expanded true leaves), baby leaf 2 (kale with six fully expanded true leaves), and adult kale (kale with eight fully expanded true leaves) were in the following order: baby leaf 1 = adult kale > baby leaf 2 > microgreen 1 > microgreen 2 [22]. Furthermore, kale can be a good source of gallic acid, catechin, epicatechin, ferulic acid, quercetin, and sinapic acid [23,24].

According to carotenoid biosynthesis gene expression and carotenoid HPLC analysis results, white-LED light treatment increased the transcriptional levels of *BoPDS* and *BoZDS* and the accumulation of most carotenoids, such as lutein, 13-*cis*-β-carotene, α-carotene, β-carotene, and 9-*cis*-β-carotene, compared with blue- or red-LED-light treatment. This result is consistent with those of the previous studies. For example, Kim and Park [25] reported that 1-month-old plantlets of red Chinese cabbage (*Brassica rapa* ssp. *pekinensis*) and green Chinese cabbage exposed to white-LED lights contained higher levels of most carotenoids than those exposed to blue- or red-LED lights [25]. Frede et al. [26] reported that white-LED irradiation increased the production of lutein and β-carotene in pakchoi (*B*. *rapa* ssp. *chinensis*) sprouts compared with blue or red-LED-light treatment, even though white- or blue-LED treatment caused high transcriptional induction of carotenoid biosynthesis genes, including *PSY* and *PDS*. Similarly, Frede et al. [27] described that the carotenoid concentrations were lower in pakchoi sprouts grown under blue LEDs, though higher expression levels of carotenoid biosynthesis genes and the *ELONGATED HYPOCOTYL5* (*HY5*) transcription factor activating *PSY* expression were observed. It might be due to higher transcription levels of carotenoid cleavage dioxygenase 4 (CCD4), which is an enzyme involved in carotenoid degradation, in blue-LED-irradiated pakchoi sprouts [27]. Furthermore, the enhanced production of carotenoid in pakchoi sprouts was reported to be higher in pakchoi sprouts grown under a combination of white and blue LEDs than in spouts grown under only white LEDs or a combination of white and red LEDs. In contrast, the influence of light quality (only white LEDs, a combination of white and blue LEDs, and a combination of white and red LEDs) on carotenoid production varied in the genus *Brassica* [28]. Considering the current study and previous studies, this study suggested that white-LED-irradiated kale microgreens had higher levels of carotenoids since white-LED lights consist of a blue-LED spectral portion inducing the expression of *HY5* transcription factor, and blue-LED treatment may degrade carotenoids in kale microgreens by causing the accumulation of CCD4. Thus, further studies are required to investigate the effect of different LED spectra on the carotenoid production in kale microgreens.

In this study, kale microgreens exposed to blue LEDs contained higher levels of most phenolics and total phenolic contents than those exposed to white or red LEDs. It may be assumed that the activation of HY5 transcription factor expression by blue LEDs upregulated flavonoid biosynthesis by inducing transcription of MYB transcription factors and flavonoid biosynthetic genes in kale microgreens. This hypothesis was supported by previous studies. The HY5 transcription factor has been reported to regulate flavonoid biosynthesis and accumulation under UV-B and visible light [29]. In particular, blue lights can induce the accumulation of the HY5 transcription factor. HY5 can activate MYB12, MYB75, and MYB-like domain (MYBD) transcription by binding to their promotor regions. In contrast, HY5 can repress transcription of MYB-LIKE 2 (MYBL2), acting as a transcriptional repressor of anthocyanin biosynthesis. MYB12 further induces the transcription of early flavonoid biosynthesis genes (chalcone synthase (*CHS*), chalcone isomerase (*CHI*), and flavanone 3-hydroxylase (*F3H*)), while MYB75 activates the expression of late flavonoid biosynthesis genes (dihydroflavonol-4-reductase (*DFR*), leucoanthocyanidin dioxygenase (*LDOX*), and UDP-glucose: flavonoid-3-O-glycosyl-transferase (*UF3GT*)) [29]. Therefore, blue-LED light is suitable for the production of phenolic compounds (gallic acid, catechin, ferulic acid, sinapic acid, rutin, and quercetin) in kale microgreens. These results are consistent with those of previous studies. For example, previous studies have reported that exposure to blue-LED light induces high production of phenolic compounds (gallic acid, quercetin, caffeic acid, sinapic acid, 4-hydroxybenzoic acid, and chlorogenic acid) in mustard (*Brassica juncea*) sprouts [30], phenolics (caffeic acid, (−)-epicatechin, and (+)-catechin) in canola (*Brassica napus*) sprouts [6], phenolics (4-hydroxybenzoic acid, ferulic acid, quercetin, and kaempferol) in Chinese cabbage (*B. rapa* ssp. *Pekinensis*) sprouts [31] and phenolic compounds (epicatechin, p-coumaric acid, and rutin) in kohlrabi (*Brassica*. *oleracea* var. *gongylodes*) sprouts [32]. Li et al. [33] reported that blue-LED light exposure led to a high content of total phenolics and anthocyanins in Chinese kale (*B*. *alboglabra* Bailey). In addition to *Brassica* sprouts, exposure to blue light enhances the production of phenolic compounds in *Pachyrhizus erosus* seedlings [34], *in vitro*-grown *Scrophularia kakudensists* [35], shoot cultures of *Swertia chirayita* [36], and *Glycine max* sprout [37].

The glucosinolate gene expression and HPLC analysis revealed that irradiation with white or blue-LED light slightly increased the accumulation of glucosinolates with high expression of *BoMYB28-2*, *BoMYB28-2*, and *BoMYB29* in kale microgreens. Previous studies reported that MYB28 activates expression of aliphatic glucosinolate biosynthetic genes (*MAM1*, *MAM3*, *CYP79F1*, *CYP79F2*, *CYP83A1*, *ST5b*, and *ST5c*), while MYB29 positively regulates transcription of aliphatic glucosinolate biosynthetic genes (*MAM1−3*, *CYP79F1*, *CYP83A1*, *SUR1*, *SOT17*, and *SOT18*) [38,39,40,41]. Thus, this study suggested that white LEDs, which are composed of a blue-LED spectral portion, and blue LEDs induced higher expression of *BoMYB28* and *BoMYB29* and then led to relatively large amounts of glucosinolates by activating transcription of aliphatic glucosinolate biosynthetic genes. This hypothesis has been supported by a previous study reporting that blue-LED treatment induced glucoraphanin production through upregulation of aliphatic glucosinolate biosynthetic genes (*CYP79F1* and *CYP83A1*) [42]. However, although irradiation with white or blue-LED light slightly increased the accumulation of glucosinolates, only minor differences were observed. These findings agree with those of previous studies reporting that only minor changes in glucosinolates were observed in mustard sprouts treated with different LED lights (white, blue, and red) [30] and that the sum of individual glucosinolates quantified in canola sprouts exposed to different LED lights (white, blue, and red) did not statistically differ [6]. However, Lee et al., described that blue-LED treatment increased glucosinolate production in sprouts of Kimchi cabbage cultivar ‘Chun Gwang’ (*Brassica campestris* cv. Chungwang), but red LEDs were suitable for glucosinolate production in sprouts of *B*. *campestris* cv. CR Ha Gwang [43].

Kale microgreens grown under three different LED light treatments exhibited a wide range of antibacterial effects, including those of *B*. *cereus* (KCTC 3624), *E*. *coli* (KCTC 1682), *E.Coli* (PVC19), *P*. *aeruginosa* (KCCM 11803), *S*. *aureus* (KCTC 3881), *M*. *luteus* (KCTC 3063), *S*. *epidermidis,* and *P*. *aeruginosa* (1113), *P*. *aeruginosa* (1828), *P*. *aeruginosa* (1731), *P*. *aeruginosa* (0225), *P*. *aeruginosa* (0826), *P*. *aeruginosa* (1378), and *P*. *aeruginosa* (p01827). Although previous studies have reported the antimicrobial effects of kale leaves and seeds [10], few have reported the antimicrobial activity of extracts from kale microgreens grown under LED light. In this study, blue-LED light was the most effective for antibacterial effects because blue-LED-irradiated microgreens showed stronger effects against pathogens. In particular, the extract of kale microgreens irradiated with blue-LED treatment only inhibited *S*. *epidermidis* growth compared to microgreens irradiated with red- and white-LED lights. This may be due to the higher concentrations of phenolic compounds. These findings were consistent with those of previous studies. For example, kale leaf and seed extracts show efficient antibacterial properties against *E. coli* (ATCC 35218) and *S. aureus* (ATCC 25923) [2], and kale microgreen extract inhibits the growth of *E. coli.* [44]. Kale leaves and petioles exhibit antibacterial effects against *E. coli, P. aeruginosa*, and *S. aureus* [45] as well as kale leaves reveal antibacterial effects against *S. aureus*, *Enterococcus faecalis*, *Bacillus subtilis* and *Moraxella catarrhalis* [46]. In addition, Chinese kale (*B*. *oleracea* var. *alboglabra*) shows antimicrobial effects against *B*. *cereus*, *B*. *subtilis*, *Streptococcus faecalis*, *S. aureus*, *Listeria monocytogene*, *P*. *aeruginosa*, *Enterobacter aerogene*, *E*. *coli*, *Salmonella serovar*, *Shigella sonnei*, and *Candida albicans* [47]. However, this is the first study on the positive effects of kale microgreens on multidrug-resistant pathogens such as *P*. *aeruginosa* (1113, 1828, 1731, 0225, 0826, 1378, and p01827).

## 5. Conclusions

In conclusion, this is the first study to quantify the glucosinolates, phenolics, and carotenoids in kale microgreens irradiated with various LED lights. Briefly, white-LED-light irradiation enhanced the carotenoid accumulation with an increase in the transcriptional levels of *BoPDS* and *BoZDS* as well as white or blue-LED-light treatment, which slightly increased the glucosinolate production with high transcript levels of *BoMYB28-2*, *BoMYB28-2*, and *BoMYB29* in kale microgreens. Additionally, blue-LED-light exposure led to high levels of phenolic compounds. The effect of light quality on glucosinolate biosynthesis in *Brassica* plants is not clearly understood yet. Therefore, it is necessary to investigate the molecular mechanisms by which light regulates glucosinolate biosynthesis. Moreover, this study comprehensively indicated a correlation between secondary metabolites and their biological activities (antioxidant and antibacterial effects). Extracts from kale microgreens exposed to blue-LED light showed greater antibacterial and antioxidant effects than those of kale microgreens exposed to other LED lights. This might be due to the higher levels of phenolic compounds. In particular, this study highlighted that the extracts had antibacterial properties against multidrug-resistant *Pseudomonas aeruginosa*. Thus, this study suggests that blue-LED lights are suitable for producing secondary metabolites in kale microgreens, and according to the bioactive compound analysis, kale microgreens can be used to treat infections and diseases caused by microorganisms.

## Figures and Tables

**Table 1:** Carotenoid contents (μg/g dry weight) of kale microgreens grown under different LED light illumination.

	White	Red	Blue
Lutein	1070 ± 10.7 a *	1080 ± 71.0 a	965 ± 6.35 b
13-*cis*-β-Carotene	153 ± 0.0780 a	106 ± 3.87 c	117 ± 7.23 b
α-Carotene	31.5 ± 0.232 a	25.0 ± 0.516 c	28.8 ± 1.59 b
β-Carotene	1980 ± 10.2 a	1680 ± 88.4 c	1850 ± 52.3 b
9-*cis*-β-Carotene	120 ± 3.85 a	96.7 ± 4.31 b	105 ± 5.16 b

* Means with different letters (a–c) are significantly different at *p* < 0.05 using DMRT.

**Table 2:** Alipathilucosinolate contents (μmol/g dry weight) of kale microgreens grown under different LED light illumination.

	White	Red	Blue
Glucoiberin	0.357 ± 0.0431 a *	0.359 ± 0.00507 a	0.298 ± 0.0248 b
Progoitrin	27.3 ± 0.119 a	23.9 ± 0.614 b	29.1 ± 2.13 a
Glucoraphanin	0.889 ± 0.0146 b	0.737 ± 0.0304 c	1.01 ± 0.0662 a
Sinigrin	0.0913 ± 0.00819 a	0.0644 ± 0.00233 b	0.0993 ± 0.0123 a
Glucobrassicanapin	0.271 ± 0.00952 a	0.240 ± 0.00282 b	0.274 ± 0.0235 a
Glucoerucin	0.700 ± 0.111 ab	0.816 ± 0.0283 a	0.615 ± 0.0288 b

* Means with different letters (a–c) are significantly different at *p* < 0.05 using DMRT.

**Table 3:** Phenolic contents (μg/g dry weight) of kale microgreens grown under different LED light illumination.

	White	Red	Blue
Gallic acid	14.1483 ± 0.3128 c *	17.8989 ± 1.773 b	75 ± 0.1472 a
Catechin	72.1758 ± 1.7539 b	68.4374 ± 1.4468 c	90.8036 ± 1.9068 a
Ferulic acid	1.8538 ± 0.1143 b	1.2222 ± 0.1239 c	1.7846 ± 0.0677 a
Sinapic acid	16.4898 ± 3.6659 b	7.2225 ± 1.7981 c	14.9793 ± 0.7898 a
Rutin	134.9817 ± 0.7248 a	141.8442 ± 6.2921 a	136.1821 ± 0.5638 a
Quercetin	112.4698 ± 0.0742 b	110.4018 ± 0.0645 b	114.8303 ± 2.0245 a

* Means with different letters (a–c) are significantly different at *p* < 0.05 using DMRT.

**Table 4:** Total phenolic content (TPC) and 2-diphenyl-1-picrylhydrazyl (DPPH) assay of kale microgreens grown under different LED light illumination.

	White	Red	Blue
TPC [mg gallic acid equivalent (GAE)/g Dry weight]	83.32 ± 3.35 b *	73.06 ± 5.58 c	93.39 ± 0.84 a
DPPH (Inhibition%)	88.16 ± 1.42 ab	87.19 ± 1.42 b	90.50 ± 0.73 a

* Means with different letters (a–c) are significantly different at *p* < 0.05 using DMRT.

## Data Availability

Not applicable.

## Supplementary Materials

The following supporting information can be downloaded at: https://www.mdpi.com/article/10.3390/antiox12091686/s1, Table S1. qRT-PCR reaction condition method.; Table S2. Primers for qRT-PCR reaction.; Table S3. Gene information used in this study.; Table S4. Antibacterial activities of methanol extracts of kale microgreens grown under different LED light illumination.; Figure S1. HPLC chromatograms.; Figure S2. LED light spectrums used in this study.
