# Supplementary material for: LED Lights Influenced Phytochemical Contents and Biological Activities in Kale (Brassica oleracea L. var. acephala) Microgreens
Source: Antioxidants (Basel). 2023 Aug 29;12(9):1686. doi: 10.3390/antiox12091686 (PMC10525181; doi:10.3390/antiox12091686)

Table S1. qRT-PCR reaction condition method.

Equipment	CFX96 real-time system with a C1000 thermal cycler (Bio-Rad, Hercules, CA)
qRT-PCR reaction condition method.	10 min at 95 °C (pre-denaturation), followed by 40 cycles of 15 s at 95 °C (denaturation), and 60 s at 60 °C (annealing and extension). All the PCR-runs were carried out in three biological and technical replicates.

Table S2. Primers for qRT-PCR reaction.

Primer name	Sequence (5′- 3′)
RT-BoGAPDH (F)	CACTATGCACCCTGCTGTTAACTG
RT-BoGAPDH (R)	TGCATCTCTGCCCACTGTAGG
RT-BoMyb28-1 (F)	CATGGACCATTGAGGAAGACAAG
RT-BoMyb28-1 (R)	ATGGAGCATGATGATAATCTGTTCC
RT-BoMyb28-2 (F)	TATCATCATGCTTCATGCTTCTCG
RT-BoMyb28-2 (R)	GGGAATGCAAATTCTCAGGTACAG
RT-BoMyb28-3 (F)	ATGCATCTAGTTCCGACAAGCG
RT-BoMyb28-3 (R)	TGATGGTAGTGGCCTTAGCAGC
RT-BoMyb29 (F)	GGACCATCGAAGAAGACAAGAAAC
RT-BoMyb29 (R)	AGCGTGAAGCATGATGATAATCTG
RT-BoPSY (F)	ATCAGAGACTCAACACT
RT-BoPSY (R)	CAGATCATCAAGGTC
RT-BoPDS (F)	ATGTTCTACCAGCACCCTTAAACG
RT-BoPDS (R)	CTCATCAGTCACGCGATCAGG
RT-BoZDS (F)	ACTCAAGCCCTATGACAAGCTGAG
RT-BoZDS (R)	AACCGAGTGCATAAGCAACAGG

Table S3. Gene information used in this study

***Brassica oleracea* ID**	**Gene description**
Bo7g098590	myb domain protein 28-1
Bo9g014610	myb domain protein 28-2
Bo2g161590	myb domain protein 28-3
Bo3g004500	myb domain protein 29
Bol021326	phytoene synthase
Bo4g127210	phytoene desaturase
Bo5g146930	ζ-carotene desaturase
Bo4g154410	NADP-dependent glyceraldehyde-3-phosphate dehydrogenase

Table S4. Antibacterial activities of methanol extracts of kale microgreens grown under different LED light illumination.

Bacterial strains	Zone of inhibition (mm)		
	**Extracts from kale microgreens irradiated with white LEDs**	**Extracts from kale microgreens irradiated with red LEDs**	**Extracts from kale microgreens irradiated with blue LEDs**
B. cereus (KCTC 3624)	11.5-12	12.5-13	13.5-14
E. coli (KCTC 1682)	9.5-10	9.5-10	10.5-11
E. Coli (PVC19)	12.0-12.5	12.5-13	14.5-15
P. aeruginosa	13.5-14	13.5-14	14.5-15
S. aureus (KCTC 3881)	11.0-11.5	11.5-12	10.5-11
M. luteus (KCTC 3063)	12.5-13	12.5-13	15-15.5
S. epidermidis	—[1]	10-10.5	—
P. aeruginosa (1113)	11.5-12	12.5-13	13.5-14
P. aeruginosa (1828)	11.5-12	11-11.5	12.5-13
P. aeruginosa (1731)	12-12.5	12-12.5	13-13.5
P. aeruginosa (0225)	12-12.5	12-12.5	12.5-13
P. aeruginosa (0826)	12-12.5	12.5-13	12.5-13
P. aeruginosa (1378)	12-12.5	12.5-13	12.5-13
P. aeruginosa (01827)	12.-12.5	12-12.5	12.5-13

[1] negative

Figure S1. HPLC chromatograms.

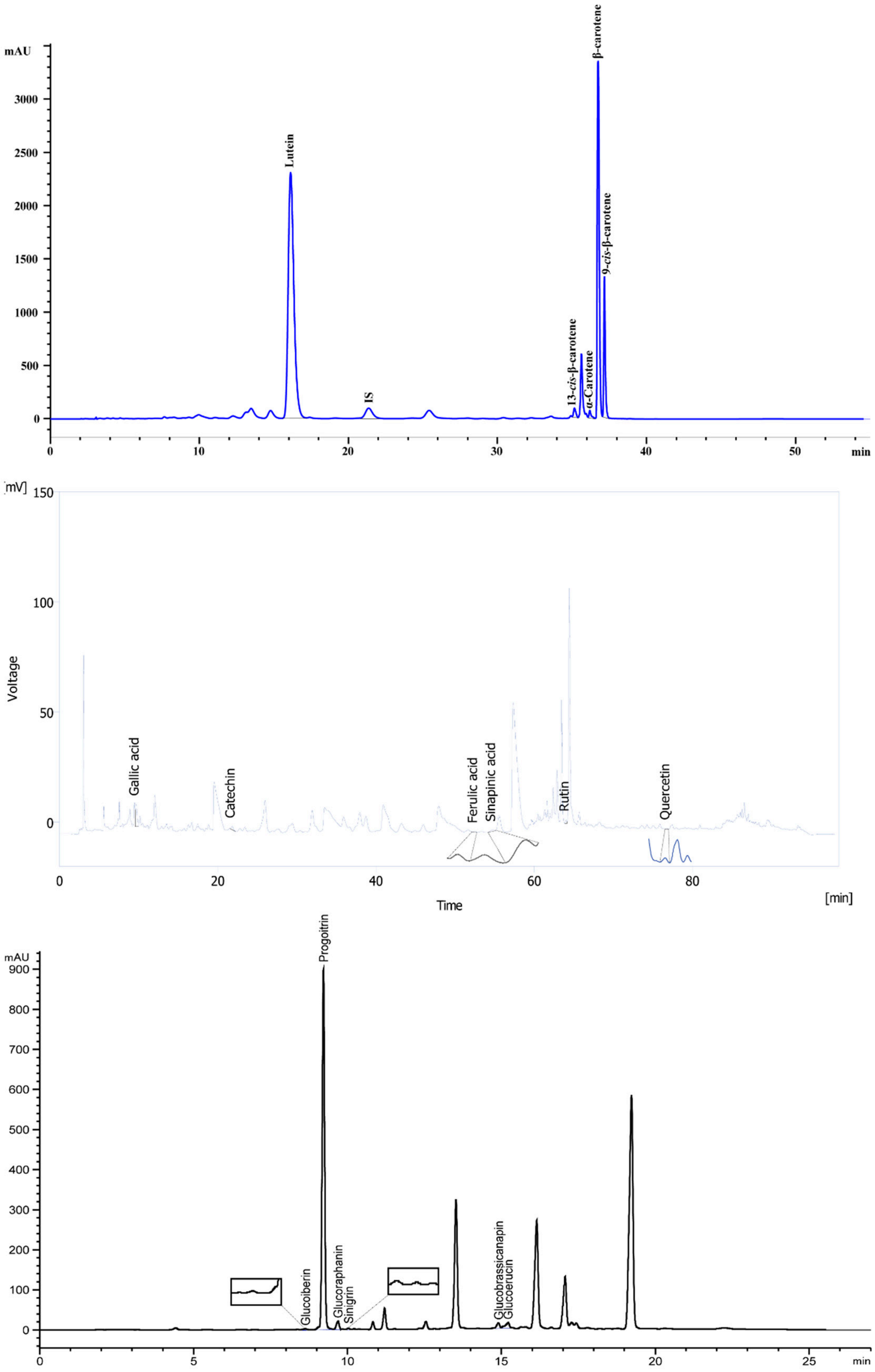

Figure S2. LED light spectrums used in this study.

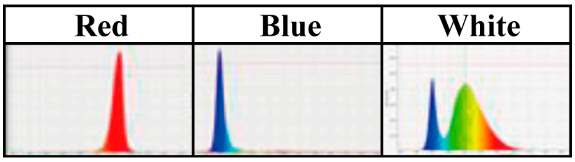

Supplement: Supplementary file 1 [file antioxidants-12-01686-s001.zip › antioxidants-2563751-supplementary.pdf]
